# Investigating the effects of home-based rehabilitation after intensive inpatient rehabilitation on motor function, activities of daily living, and caregiver burden

**DOI:** 10.1371/journal.pone.0316163

**Published:** 2024-12-27

**Authors:** Kenji Sato, Eri Otaka, Kenichi Ozaki, Kenta Shiramoto, Rie Narukawa, Takeshi Kamiya, Masaki Kamiya, Daiki Shimotori, Chiaki Kamizato, Naoki Itoh, Hitoshi Kagaya, Izumi Kondo

**Affiliations:** 1 Department of Rehabilitation Medicine, National Center for Geriatrics and Gerontology, Obu, Aichi, Japan; 2 Laboratory of Practical Technology in Community, Assistive Robot Center, National Center for Geriatrics and Gerontology Research Institute, Obu, Aichi, Japan; 3 Assistive Robot Center, National Center for Geriatrics and Gerontology Research Institute, Obu, Aichi, Japan; University of the Witwatersrand Johannesburg, SOUTH AFRICA

## Abstract

**Background:**

Home-based rehabilitation involves professional rehabilitation care and guidance offered by physical, occupational, and speech therapists to patients in their homes to help them recuperate in a familiar living environment. The effects on the patient’s motor function and activities of daily living (ADLs), and caregiver burden for community-dwelling patients are well-documented; however, little is known about the immediate benefits in patients discharged from the hospital. Therefore, we examined the effects of continuous home-based rehabilitation immediately after discharge to patients who received intensive rehabilitation during hospitalization.

**Methods:**

We retrospectively reviewed 150 patients [mean (standard deviation, SD) = 81 (9) years] discharged from the convalescent rehabilitation and community-based integrated care wards undergoing tailored home-based rehabilitation for 6 months (provided by physical or occupational therapists: 1–2 sessions of 40–60 min each per week). The outcome measures at baseline and after 3 and 6 months were compared.

**Results:**

The participants included in this study had orthopedic (n = 76), cerebrovascular (n = 50), neuromuscular (n = 11), cardiovascular (n = 5), respiratory (n = 3), cancer (n = 3) and other diseases (n = 2). The mean (SD) time from discharge to the start of rehabilitation was 4 (4) days. One-way analysis of variance and post-hoc comparisons showed significant improvements at 3 months from baseline in grip strength (p = 0.002), 5-repetition sit-to-stand test (p < 0.001), Standing test for Imbalance and Disequilibrium test (p = 0.025), Functional Independence Measure (p < 0.001), modified Frenchay Activities Index (p < 0.001). Additionally, a statistically significant improvement was observed in the Japanese Zarit Caregiver Burden Interview score at 6 months from baseline (p < 0.001).

**Conclusions:**

Home-based rehabilitation improves motor function, ADLs, and instrumental ADLs even after intensive inpatient rehabilitation and decreases the burden of the caregiver in the long term. Hence, tailored home-based rehabilitation should be continuously implemented after the completion of intensive inpatient rehabilitation.

## Introduction

Home-based rehabilitation (HBR) is an important component of providing continuing care in a familiar living environment [1]. Essentially, physical therapists, occupational therapists, and speech therapists visit the patient’s home and provide rehabilitation services. HBR facilitates the patient’s resumption of daily activities, improve quality of life, prevent the development of new disabilities by supporting what the patient desires, and reduce caregiver burden. Systematic reviews have shown that HBR is effective in maintaining and improving basic activities of daily living (ADLs) in various diseases, such as stroke [2, 3], femoral neck fracture [4, 5], and neuromuscular diseases [6]. However, the onset of diseases or time from hospitalization vary in these reports. Some studies supported the usefulness of HBR as part of early supported discharge [7, 8]. Since a decrease in physical activity after discharge is a risk factor for subsequent decline in physical function, it is particularly important to provide rehabilitation that encourages activity immediately after discharge [9–12].

Japan has a specialized medical system for rehabilitation [13], a notable component of which is the “convalescent rehabilitation ward” where intensive inpatient rehabilitation is provided for patients in the subacute stage [14]. Patients who satisfy the requirements can be admitted to this ward for 90–180 days and receive up to 3 h of rehabilitation per day using medical insurance [13]. Likewise, “community-based integrated care wards” offer intensive inpatient rehabilitation for patients who have problems discharging home after acute care [15]. Similarly, patients who satisfy the requirements can be admitted to this ward for 60 days and receive up to 3 h of rehabilitation per day using medical insurance as well. HBR can be provided under both systems; however, the benefits of continued HBR after intensive inpatient rehabilitation programs are yet to be elucidated.

The main advantage of HBR over inpatient rehabilitation is that evaluation and training for activities can be practiced in the patients’ real-life environment [31]. Given that the performance of daily activities can be influenced by the environment, some activities that could be performed independently in a hospital may not be performed similarly at home. Conversely, some activities that require assistance in the hospital environment can be achieved through environmental adjustments and repetitive training at home. Moreover, some studies have reported that certain activities that could be trained during inpatient rehabilitation are assisted by caregivers after discharge because they are time-consuming and unsafe, thereby decreasing the patient’s independence in performing ADLs (e.g., bathing, dressing, walking, and stair climbing) [16, 17]. HBR provides the opportunity to discuss solutions to these activities with caregivers to maximize patient safety, independence, and efficiency in real-life situations. This factor can be considered another advantage of HBR that is difficult to achieve through inpatient rehabilitation. These features of HBR are presumed to remain significant even after intensive inpatient rehabilitation. Therefore, research verifying whether HBR helps mitigate the activity barriers associated with this transition to the home environment and maintains or improves the physical functions and ADLs acquired during inpatient rehabilitation is necessary. Accordingly, this study aimed to determine the effectiveness of HBR immediately after discharge from the hospital in patients undergoing intensive inpatient rehabilitation.

## Materials and methods

### Study design and participants

This retrospective study was conducted at the National Center for Geriatrics and Gerontology (NCGG), Aichi, Japan after obtaining approval from the local Ethics Review Committee (No. 1582–2). Medical records of outpatients who had received HBR from the NCGG between June 2016 and September 2022 were screened. Of these, individuals who were discharged from the convalescent rehabilitation ward and community-based integrated care ward of the hospital and had started HBR within one month after discharge which continued for at least six months were considered for inclusion into the study. Patients who could not be regularly assessed for motor function, ADL evaluation, and whose caregivers could not be evaluated for the burden of care during the period of HBR due to hospitalization or other reasons were excluded from the study. Regarding informed consent, opt-out consent procedures were adopted wherein the study protocol was disclosed via the hospital’s website and posters within the hospital to provide the patients with the opportunity to refuse participation.

### Home-based rehabilitation program

Physical and occupational therapists provided the HBR program. Each patient received 1–2 sessions per week on average (40–60 min/session) for at least 6 months. At the beginning of each visit, vital signs, pain, edema, appetite, sleep status, and recent falls were assessed. Subsequently, each patient was provided with a personalized HBR program. Each program invariably provided functional training, promoted practice of ADLs and instrumental ADLs (IADLs), provided information and instruction regarding the use of assistive devices, encouraged social participation and setting further rehabilitation goals, provided instruction on daily exercises, and provided caregiver support (Table 1), with the proportion of time spent on each item varying depending on the patients’ characteristics and needs. The program was organized based on each patient’s goals and action plan, as assessed by the Canadian Occupational Performance Measure (COPM) [18], in collaboration with the physician and care manager.

**Table 1:** Home-based rehabilitation program.

Item	Concrete example
Functional training	Includes resistance training, aerobic exercise, mobilization, stretching, balance and weight bearing, etc.
ADL and IADL exercises	ADL exercises: hands-on exercises, such as transferring, walking, climbing stairs, eating, toileting, grooming, dressing, and bathing; IADL exercises: hands-on exercises, such as meal preparation, dishwashing, laundry, housework, and going out.
Suggestions for using walking aids and assistive devices	Suggestions for the elimination of environmental hazards by using stepladders or slopes, using assistive devices, such as nursing beds, shower chairs, chairs for putting on and taking off shoes, as well as walking aids for indoor and outdoor use, and advice on home modifications, such as installing handrails near steps and doors.
Supporting social participation and setting rehabilitation goals	Setting rehabilitation goals using the Canadian Occupational Performance Measure (COPM) and encouraging reengagement in valuable activities, such as social interaction and participation in domestic activities, through motivational interviewing and other methods.
Multidisciplinary cooperation	Cooperate with multiple departments, including geriatrics, orthopedics, neurology, and cardiology, to share rehabilitation goals, progress, and symptom changes with each department, Additionally, to consult with them about adjustments to medications and therapeutic orthotics to suit the patient’s condition.
Guidance on daily physical activity	Includes providing an exercise program appropriate to the patient’s ability and monitoring their daily exercise status.
Carers support	Includes teaching caregiving practice and actively listening to the caregiver’s care burden.

ADLs, Activities of daily living; IADLs, Instrumental activities of daily living

### Assessment of outcome measures

Basic characteristics collected from medical records included age, gender, disease, Japanese version of the Mini-Mental State Examination (MMSE-J), time from injury or disease onset to discharge from the hospital (hospitalization period), time from discharge to the start of HBR, and the number of people living with the patient. The data were accessed for research on December 23, 2022. The authors did not access any personally identifiable participant information during or after data collection.

The primary outcome was instrumental ADLs assessed using the Frenchay Activities Index (FAI) [19, 20], which consists of 15 items on ADLs, including social activities [0 (most inactive) to 45 (most active)]. The secondary outcomes included basic ADLs assessed using the 18-item Functional Independence Measure (FIM) [each scored 1 (severely dependent) to 7(independent), with total scores ranging 7–135] [21–23]; motor function assessed using grip strength [24]; the 5-repetition sit-to-stand test (5-rep STS test) [25] for muscle strength and Standing test for Imbalance and Disequilibrium (SIDE) for postural balance ability [0 (unable to stand), 1, 2a, 2b, 3, 4 (able to stand on one foot)] [26]; and caregiver burden measured using the 22-item Zarit Caregiver Burden Interview (J-ZBI) [0 (lightest) to 88 (heaviest)] [27, 28]. These outcome measures were assessed at baseline and after 3 and 6 months.

### Statistical analysis

Scores for each indicator were compared at the three time points using repeated measures one-way ANOVA for continuous variables and the Friedman test for ordinal variables. Two-tailed Student’s t-test and Wilcoxon signed rank test were used for post-hoc analysis, respectively, with Bonferroni adjustment to correct for p-values (p-values were multiplied by the number of tests). All statistical analyses were performed using SPSS (version 26; IBM Corp., Armonk, NY, USA). Statistical significance was set at p < 0.05.

## Results

A total of 150 patients met the eligibility criteria (mean (SD) age = 81 (9) years; 74 (49.3%) were male). The mean (SD) MMSE-J score of the study participants was 23.8 (7.2). On average, the length of hospitalization was 102 (45) days, while the mean time between discharge and the start of HBR was 4 (4) days. Orthopedic and cerebrovascular diseases were the most common diseases among participants (Table 2).

**Table 2:** Baseline characteristics of study participants.

		Mean ± SD	%
Age (y)		81 ± 9	
Gender	Male (%)		49.3
Primary disease	Bone and joint disease (%)		50.7
	Cerebrovascular disease (%)		33.3
	Neuromuscular disease (%)		7.3
	Cardiovascular disease (%)		3.3
	Respiratory disease (%)		2.0
	Cancer (%)		2.0
	Others (%)		1.3
Hospitalization (days)		102 ± 45	
	Bone and joint disease	92 ± 42	
	Cerebrovascular disease	116 ± 45	
	Neuromuscular disease	125 ± 51	
Time from discharge to start of home-based rehabilitation (days)		4 ± 4	
Number of households	1 (%)		19.5
	2 (%)		47.6
	3 or more (%)		32.9
Cognitive function	MMSE-J	23.8 ± 7.2	

SD, standard deviation; MMSE-J, Mini Mental State Examination-Japanese

Regarding motor function, statistically significant differences were observed for all outcome measures (grip strength, 5-rep STS test, and SIDE) measured at the three time points (grip strength: p = 0.001; 5-rep STS test: p < 0.001; SIDE: p < 0.001). Further multiple comparison results showed that statistically significant improvements were seen at both 3 and 6 months compared to the baseline (grip strength: p = 0.002 and p = 0.006; 5-rep STS test: p < 0.001 and p < 0.001; SIDE: p = 0.025 and p = 0.004, respectively) (Table 3).

**Table 3:** Comparisons of outcomes after three and six months of home-based rehabilitation.

	Mean (SD), Median [IQR]				Repeated measures analysis of variance		Post-hoc tests		
	Baseline	After 3Mo.	After 6Mo.				3 Mo. vs Baseline	6 Mo. vs Baseline	6 Mo. vs 3 Mo.
				N	F (χ^2^)	p-value	p-value	p-value	p-value
Grip strength (kgf)	18.4 (8.0)	19.2 (7.8)	19.3 (8.2)	147	8.3	.001	.002	.006	1.000
5-rep STS test (sec)	19.2 (9.5)	15.6 (7.4)	15.1 (7.6)	139	40.2	< .001	< .001	< .001	.589
SIDE	2a [2a–3]	2b [2a–3]	2b [2a–3]	149	(26.7)	< .001	.025	.004	1.000
FIM (pt)	105 [84–115]	108 [93–118]	109 [95–118]	150	(94.1)	< .001	< .001	< .001	.121
FAI (pt)	5 [0–10]	11 [5–20]	11 [6–20]	142	(113.2)	< .001	< .001	< .001	.290
J-ZBI (pt)	18 [9–28]	13 [5–28]	11 [5–27]	123	(17.0)	< .001	.071	< .001	.273

5-rep STS test, Five-repetition sit-to-stand test; SIDE, Standing test for Imbalance and Disequilibrium (1, 2, 3, and 4 indicate SIDE Ⅰ, Ⅱa, Ⅱb, and Ⅲ, respectively); FIM, Functional Independence Measure; FAI, Frenchay Activities Index; J-ZBI, Japanese version of the Zarit Caregiver Burden Interview; SD, standard deviation; IQR, interquartile range; n = number of participants; F = F-value; χ2 = chi-square.

Likewise, the measures for ADLs and IADLs (FIM and FAI, respectively) showed statistically significant differences between values for the three time points (FIM: p < 0.001; FAI, p < 0.001). Multiple comparison tests showed improvement in both FIM and FAI at both 3 and 6 months compared to the baseline (FIM: p < 0.001 and p < 0.001; FAI: p < 0.001 and p < 0.001; Table 3). Lastly, the caregivers’ care burden score (J-ZBI) also showed a statistically significant difference between the three time points (p < 0.001), particularly at 6 months after initiating HBR (p < 0.001; Table 3).

## Discussion

This study retrospectively analyzed changes in the motor function and independence in ADLs of patients who have undergone 6 months of HBR after discharge from the convalescent and the community-based rehabilitation wards, and the effects of this intervention on the care burden of their caregivers. At 3 months after initiation, all items, except J-ZBI, showed statistically significant improvement, and by 6 months of HBR, all outcome measures showed improvement. These results highlight that the continuation of HBR immediately after discharge is valuable in promoting physical and functional independence in patients with various cerebrovascular, osteoarticular, and neuromuscular diseases, and eventually reduces the care burden on their caregivers.

### Impact on motor function

We observed significant improvements in all measures of motor function after 6 months of HBR, which concurs with the results of previous studies on elderly people living in the community [29, 30]. HBR, including functional training of the upper and lower limbs and postural balance exercises for 40 weeks, in stroke patients at least 6 months after stroke onset, led to significant improvements in the 5-rep STS test and Tinetti’s Balance Scale [29]. In another study, physical therapists visited patients with hip fractures approximately 9.5 months after the injury, distributed an exercise program, asked them to record their exercise, and conducted regular monitoring for 6 months [30]. They noted improvements in the Short Physical Performance Battery and the Activity Measure for Post-Acute Care, both of which assess motor function and mobility. The participants included in this study had a shorter post-onset/injury period than those in the aforementioned studies. Given that motor recovery is generally more evident in the early post-onset/injury period, it can be reasonably assumed that continued HBR would promote improvement in motor function. Notably, the current study showed that the effects of HBR on motor function persist after intensive inpatient rehabilitation, during which the maximum possible extent of rehabilitation is provided. Presumably, encouraging frequent performance of exercises in daily life, including days where the therapist does not visit—an essential component of the HBR program—may effectively improve motor function.

### Impact on ADLs and IADLs

Significant improvements in independence in ADLs and IADLs were observed, which began as early as 3 months after initiating HBR. Similar findings have been reported in previous studies on the effects of HBR on ADLs and IADLs for chronic patients which advocate for tailored rehabilitation programs designed according to the patient’s condition and goals [2, 4, 31–33]. The present study further corroborates that patients can regain independence in ADLs earlier with HBR immediately after discharge. This result can be attributed to the HBR program designed according to the patient’s condition and goals. Environmental adjustments, such as the installation of nursing beds, shower chairs, chairs for dressing and undressing shoes, handrails near steps and doors, evaluation and suggestion of walking aids, and reduction of steps and thresholds in the room are some of the characteristic features of HBR that cannot be attained only with inpatient rehabilitation [34]. Therefore, these interventions tend to facilitate functional recovery even in patients who have undergone intensive inpatient rehabilitation.

### Impact on caregiver burden

Another important effect of HBR is the reduction in the caregiver burden. Our findings showed an improvement in the J-ZBI score after 6 months of HBR. Although similar results have been reported with HBR programs for community-dwelling stroke patients [31], the current study emphasizes that these positive effects of HBR from the caregivers’ perspective can be attained soon after discharge from inpatient rehabilitation. Two key aspects facilitate these results. First, improvements in the patient’s motor function through functional training and environmental adjustments implemented during HBR enable early independence in ADLs and increase the frequency of participation by the patients [31, 35]. Second, family guidance provided to caregivers reinforce positive attitudes when dealing with daily problems, as is observed with patient education and motivational interviewing [36].

### Benefits of HBR program early after discharge from the hospital

This study draws attention to the favorable effects of initiating HBR promptly after discharge from the hospital for patients who received inpatient rehabilitation. Notably, even after receiving inpatient rehabilitation for approximately 3 months, HBR immediately after discharge helped regain functional recovery. Given that previous studies have reported functional decline in the medium to long term after discharge [12, 16, 37], our findings carry tremendous significance for patients’ lives after discharge. Overall, our results showed that implementation of HBR early after discharge could be effective for two reasons. First, making environmental adjustments and sharing rehabilitation goals and progress with family members could help patients cope with “over-care by caregivers” and “refraining from activities to prevent falls,” which are the primary reasons for the decline in ADLs [16]. Second, evidence has shown that patients cannot completely regain their pre-onset/injury functional state even after intensive inpatient rehabilitation [38, 39]. This could be possibly considered as a potential for further improvement. Therefore, continuous HBR among patients who could not be sufficiently convalesced during hospitalization may have promoted functional recovery, which ultimately reduced caregiver burden.

The study had certain limitations. First, according to our hospital’s protocol, it was not possible to have a control group that did not receive HBR, so the results could not be compared with the natural follow-up after discharge from the hospital. Second, the exact number of total HBR visits or sessions on certain activities (e.g., functional training or ADL training) per participant could not be obtained from the existing data. Hence, future studies should analyze the relationship between the total time spent on a specific training and its effectiveness. Lastly, because the study was conducted at a single hospital, potential bias due to the hospital’s environment and characteristics cannot be ruled out. Nevertheless, this study offers clinically meaningful results to verify the effects of continuous HBR immediately after discharge. The results of this study suggest that immediate and subsequent HBR should be provided even after intensive inpatient rehabilitation. The next step would be to identify the characteristics of patients who are more or less likely to benefit from HBR.

## Conclusion

A continuous custom-tailored HBR program immediately after discharge for patients who underwent intensive inpatient rehabilitation helps retain and improve motor function and independence in ADLs and IADLs, ultimately reducing caregivers’ burden. This study will help support patients’ independence and reduce the burden of home and nursing care in clinical settings.

## Supporting information

S1 Data Original data. (XLSX)
